# Fermentation Process Optimization of Strawberry Wine Using the 2-Phenylethanol Tolerant *Saccharomyces cerevisiae* AFRC01 and Comparative Genomic Analysis

**DOI:** 10.3390/foods14173043

**Published:** 2025-08-29

**Authors:** Dan Liu, Zelin Yang, Xue Wang, Meng Xu, Xinqi Zhang, Tong Wu, Wenhui Wu, Shu Zheng, Yan Li, Fumeng He, Yongqing Xu, Fenglan Li, Wei Lan

**Affiliations:** 1 Anhui Engineering Research Center for Functional Fruit Drink and Ecological Fermentation, Fuyang Normal University, Fuyang 236037, China; s230902014@neau.edu.cn; 2 College of Life Sciences, Northeast Agricultural University, Harbin 150030, China; b230902001@neau.edu.cn (D.L.); b240902032@neau.edu.cn (X.W.); s220902014@neau.edu.cn (M.X.); s240901803@neau.edu.cn (X.Z.); s240902132@neau.edu.cn (T.W.); s240902128@neau.edu.cn (W.W.); s240902154@neau.edu.cn (S.Z.); s220902015@neau.edu.cn (Y.L.); hefumeng@neau.edu.cn (F.H.); xuyongqing@neau.edu.cn (Y.X.); lifenglan@neau.edu.cn (F.L.)

**Keywords:** strawberry wine, *Saccharomyces cerevisiae*, comparative genomics, 2-Phenylethanol

## Abstract

The fermentation of fresh strawberries into fruit wine offers a solution to the seasonal surplus of strawberries. 2-phenylethanol is a key compound influencing the flavor profile of fruit wines, and elevating its content can enhance both flavor complexity and body quality. In this study, we established the optimal process of fermenting strawberry wine using the 2-phenylethanol (3.9 g/L) tolerant *S. cerevisiae* AFRC01 strain. Under optimal conditions, the 2-phenylethanol content reached 170.06 mg/L, representing a 33% increase compared to the unoptimized process. Wine fermented with *S. cerevisiae* AFRC01 exhibited reduced astringency and aftertaste alongside enhanced richness and freshness compared with that fermented with *S. cerevisiae* CICC33253. A further analysis of the gene expression patterns in the 2-phenylethanol synthesis pathway under 2-phenylethanol stress (1.0 g/L) revealed no significant stress-responsive changes in *S. cerevisiae* AFRC01, suggesting its mechanism of tolerance is minimally associated with regulation of this pathway. Subsequent genome sequencing identified mutations in the glycolytic pathway genes involved in synthesizing phosphoenolpyruvate (the 2-phenylethanol precursor) in *S. cerevisiae* AFRC01 compared with *S. cerevisiae* CICC33253. This study provides a foundation for the later promotion and application of 2-phenylethanol tolerant *S. cerevisiae* AFRC01.

## 1. Introduction

Strawberry (*Fragaria ananassa*) is a perennial herbaceous plant bearing fruits with soft, juicy tissue and a distinct sweet-sour flavor [1]. It contains a variety of nutritional compounds (e.g., sugars, vitamins, and minerals) alongside non-nutritive bioactive compounds (e.g., flavonoids, anthocyanins, and phenolic acids) [2,3]. China’s strawberry production has grown significantly, ranking first globally in annual output and fifth in export volume [4]. However, fresh strawberries are highly perishable due to their susceptibility to decay, low cuticular strength and poor storability, making postharvest preservation increasingly challenging [5]. Fermentation and brewing of strawberries into fruit wine can address seasonal overcapacity of fresh strawberries, serving as an effective strategy to enhance value-added strawberry products. Additionally, strawberry wine contains abundant flavor compounds; the harmonious integration of fruity and vinous aromas appeals highly to consumers, with high market recognition [6]. Microbial fermentation represents a key process in fruit wine flavor development, offering advantages such as mild reaction conditions, environmental sustainability, and operational simplicity [7,8,9,10,11]. Among fermentative microorganisms, *Saccharomyces cerevisiae* is predominantly utilized in fruit wine production, generating abundant flavor-active metabolites during fermentation [12,13,14,15].

During fruit wine fermentation, key flavor compounds serve as the central chemical determinants of organoleptic quality. Higher alcohols constitute the most influential aroma compounds, among which 2-phenylethanol (2-PE) is the most abundant [16]. Exhibiting a pleasant rose-like aroma, 2-phenylethanol functions as a key flavor compound in fruit wines [17]. Elevating its concentration enhances sweet aromatic notes and improves overall sensory quality. Furthermore, 2-phenylethanol is widely employed for flavor modulation in fruit wine and beer due to its remarkable antioxidant properties, which delay fruit wine oxidation and preserve fresh fruity aromas [18]. Wilson et al. identified 2-phenylethanol as a key contributor to cider flavor [19]. Additionally, Wilson et al. demonstrated that 2-phenylethanol enhances cider’s sensory properties and provides potential health benefits through antioxidant activity [10]. Studies indicate that enhancing *S*. *cerevisiae* tolerance to 2-phenylethanol is critical for improving its biosynthesis yield [20]. Beyond strain tolerance optimization, key fermentation parameters (e.g., temperature, inoculum size, and sugar concentration) significantly influence the organoleptic properties of strawberry wine [21,22]. Tian et al., significantly enhanced volatile compounds and sensory quality in grape wine by optimizing fermentation temperature, material-liquid ratio, and initial Brix [23]. Similarly, Wilson et al. enhanced the 2-phenylethanol content in cider from 20–50 mg/L to 150–300 mg/L through strain modification and process optimization [10]. Notably, higher alcohols below 300 mg/L impart desirable aromatic complexity, whereas concentrations exceeding 400 mg/L produce off-flavors and harsh aftertastes [24,25]. Thus, selecting *S. cerevisiae* strains with favorable fermentation characteristics and optimizing process parameters is essential for premium strawberry wine production.

Currently, genomic analysis is widely employed to investigate adaptive responses to environmental stressors (e.g., osmotic pressure and elevated temperatures) in diverse organisms [26]. Comparative genomic approaches elucidate the microbial adaptation mechanisms and identify the core metabolic pathways governing product biosynthesis [27]. For instance, an analysis of the genome of the *S. cerevisiae* HJ01 revealed that the biosynthesis of aromatic alcohols such as 2-phenylethanol is mainly based on the Ehrlich pathway [28]. Comparative genomics elucidates gene functions, expression mechanisms, and associated features by examining structural variations across genomes [29,30]. Recent studies have compared *Clostridium* genomes from fermentation pit mud habitats and non-pit habitats, revealing niche-specific adaptations [31]. Thus, comparative genomic analysis provides a robust framework for investigating adaptive evolution in *S. cerevisiae* under high 2-phenylethanol stress conditions.

Studies indicate that enhancing microbial tolerance to 2-phenylethanol is critical for high yield 2-phenylethanol production [32]. MMC permits continuous subculture, enabling microorganisms to automatically undergo high-throughput adaptive evolution to obtain strains that exhibit improved growth characteristics under specific pressures [26]. To increase the commercial value of strawberries, we previously performed adaptive evolution of *S. cerevisiae* CICC33253 using the microbial microdroplet culture (MMC) system, obtaining the 2-phenylethanol (3.9 g/L)—tolerant *S. cerevisiae* AFRC01 strain. This strain was subsequently used to ferment strawberry wine. We optimized the fermentation process using 2-phenylethanol content as a key indicator to enhance the flavor profile of the wine. Concurrently, a comparative genomics analysis of *S. cerevisiae* CICC33253 and AFRC01 was conducted to preliminarily elucidate the mechanisms behind the 2-phenylethanol tolerance of *S. cerevisiae* AFRC01.

## 2. Materials and Methods

### 2.1. Experimental Materials

Fresh strawberries (purchased from Taisige Co., Ltd., Harbin, China) were washed, destemmed (calyces and pedicels removed), pat-dried with filter paper, portioned, and stored at −20 °C. *S. cerevisiae* AFRC01 was obtained through MMC system (provided by Wuxi Tmaxtree Biotechnology Co., Wuxi, China) screening of *S. cerevisiae* CICC33253 (provided by Fuyang Normal University) under high concentrations of 2-phenylethanol [26,33]. Both *S. cerevisiae* CICC33253 and AFRC01 strains were revived identically: 500 μL of yeast suspension was inoculated into 50 mL of potato dextrose water (PDW) broth and incubated at 28 °C for 48 h with 150 rpm (Shanghai 3S Technology Co., Ltd., Shanghai, China). Activated cultures were streaked onto potato dextrose agar (PDA) plates using a three-zone technique and incubated for 48 h at 28 °C. Single colonies were transferred to PDW medium and cultured for 48 h at 28 °C and 150 rpm.

### 2.2. Selection of Fermented Strawberry Wine for Single-Factor Experiments

An *S. cerevisiae* AFRC01 strain with 1% inoculum size was used under baseline conditions of 28 °C, 16 °Bx, and 8 d fermentation [34]. Experiments were conducted on four factors: fermentation temperature (22 °C, 25 °C, 28 °C, 31 °C and 34 °C), inoculation amount (0.1%, 0.5%, 1.0%, 1.5% and 2.0%), initial °Brix (12.0 °Bx, 14.0 °Bx, 16.0 °Bx, 18.0 °Bx and 20.0 °Bx), and fermentation duration (7.0, 8.0, 9.0, 10.0 and 11.0 days). Details of the protocols are provided in Table S1. After fermentation with the *S. cerevisiae* AFRC01 and *S. cerevisiae* CICC33253 strains, 2-phenylethanol content was quantified via High-Performance Liquid Chromatography (HPLC) to determine each factor’s impact.

### 2.3. Quantification of 2-Phenylethanol Content

Post-fermentation supernatants were collected via centrifugation at 4650× *g* for 10 min. After 10-fold dilution, the samples were filtered through 0.22 μm nylon membranes, with filtrates used for 2-phenylethanol quantification. An HPLC analysis was conducted using an Agilent 1260 system (Agilent Technologies, Santa Clara, CA, USA) with UV detection. Separation was performed, employing a C18 column (4.6 × 150 mm, 2.7 μm) with isocratic elution (methanol–water ratio at 55:45, *v*/*v*) at 0.5 mL/min. The detection wavelength was set at 260 nm with the column temperature maintained at 30 °C and an injection volume of 10 μL. Standard solutions of 2-phenylethanol were prepared at concentrations of 0.1 g/L, 0.2 g/L, 0.3 g/L, 0.4 g/L, and 0.5 g/L, with three replicates for each concentration. The peak areas corresponding to the different concentrations of standard solutions were determined using HPLC, and the standard curve for 2-phenylethanol was plotted based on the peak areas (Figure S1). The standard curve equation is y = 1279.4x − 0.6058, with R^2^ = 0.9994.

### 2.4. Response Surface Methodology Optimization and Model Validation

Based on the single-factor optimization results, key variables influencing 2-phenylethanol content were identified. A Box–Behnken design (BBD) was implemented to optimize three critical parameters: fermentation temperature (25, 28, and 31 °C), inoculation amount (0.1, 0.5, and 1.0%), and initial °Brix (14.0, 16.0, and 18.0 °Bx). The experimental design investigated three factors at three coded levels (−1, 0, and +1). Details of the factor-level combinations are provided in Table S2. A response surface methodology (RSM) analysis was performed using Design-Expert 13 to model the 2-phenylethanol yield and determine the optimal fermentation conditions. Model predictions were subsequently validated experimentally.

### 2.5. Sensory Analysis

Electronic tongue potential sensors have low selectivity and high cross-sensitivity, as well as high stability, making them a suitable pattern recognition method. The use of an electronic tongue system does not require complex pre-processing of the sample and enables the detection of different types of foodstuffs and the objective digital evaluation of basic taste sensory indexes of the sample, while avoiding human determination errors and achieving good repeatability [35,36]. Fermentation was carried out under the optimal fermentation process conditions, the fruit wine fermentation broth was subjected to sample pre-treatment (same method as in Section 2.3), 30 mL of the sample was added to a tasting cup, and the fermentation was examined using the electronic tongue (TS-5000Z, INSENT Co., Ltd., Kanagawa, Japan). The advantage of this device is that it is a taste analysis system that matches human taste sensory evaluation. It detects changes in membrane potential generated by electrostatic or hydrophobic interactions between various taste substances and artificial lipid membranes, without the need for statistical analysis or modeling. The electronic tongue solution was as follows: reference solution: 30 mmol/L KCl + 0.3 mmol/L tartaric acid; negative solution: water + 30% anhydrous ethanol + 100 mmol/L HCl; and positive-solution: 100 mmol/L KCl + water + 30% anhydrous ethanol + 10 mmol/L KOH. Each group of tests was performed three times. Fruit wines were tested and the taste test conditions are shown in Table S3.

### 2.6. Genome Sequencing and Functional Annotation

Single colonies of *S. cerevisiae* AFRC01 and CICC33253 were inoculated into PDW medium and activated as described in Section 2.1. Cell pellets were collected via centrifugation at 4 °C and 9500× *g* for 10 min. Genome sequencing was performed by Majorbio Co., Ltd. (Shenzhen Weike Meng Technology Group Co., Ltd., Shenzhen, China) using PacBio Sequel II and Illumina NovaSeq PE150 platforms. Raw reads were assembled with Falcon, followed by error correction using Racon [37,38]. Assembly completeness was evaluated via BUSCO using metaeuk and hmmer. Repeat Masker was used to predict dispers repeats; TRF to detect tandem repeats; Circos to generate genome visualizations; and IPRscan to conduct Gene Ontology (GO) annotation. Diamond was used to assign KEGG pathway and CAZy database annotations. Comparative genome alignments were performed with MUMmer (v3.23).

### 2.7. Expression Analysis of Phenylethanol Biosynthesis-Related Genes

We analyzed the expression patterns of genes related to the major pathways (Ehrlich and shikimate pathways) of 2-phenylethanol synthesis. *S. cerevisiae* CICC33253 and AFRC01 were added to a medium containing 1.0 g/L 2-phenylethanol, with unstressed controls. After 24 h of activation, the cells were harvested by centrifugation (1000 rpm, 10 min). Total RNA was isolated using the BayBiopure Magnetic Bead Total RNA Extraction Kit (TZRM-64-Autopure 32A; BayBiop, Guangzhou, China). RNA concentration was measured using an Implen NanoPhotometer (P330, Implen, Munich, Germany). Samples exhibiting A260/A280 ratios between 1.8 and 2.0 were selected for downstream processing. The extracted RNA was then analyzed via agarose gel electrophoresis, which revealed distinct 28S, 18S, and 5S ribosomal RNA bands, indicating high quality (Figure S2). Subsequently, reverse transcription was conducted with 1 μg RNA using the cDNA Synthesis SuperMix (AT311; TransGen Biotech, Beijing, China). Reactions were performed with ChamQ SYBR Green qPCR Master Mix II (Q711; Vazyme, Nanjing, China), using *UBC6* as the internal reference gene. Amplification efficiencies for all primer pairs were calculated from standard curves generated using quickQrtPCR v0.2.0, yielding efficiencies of 95.68–106.28% and these values fell within the optimal range (Table S4). The relative expression was calculated via the 2^−ΔΔCT^ method [39]. Primers designed with Beacon Designer 8.14 were synthesized by Comate Bioscience Co., Ltd. (Changchun, China). Details about the primers are provided in Tables S4 and S5.

### 2.8. Statistical Analysis

Each group of tests was performed three times, and the data are presented as the means ± standard deviations (means ± SDs). The significance of the differences in 2-phenylethanol content was analyzed and plotted using GraphPad Prism 8.0 with one-way ANOVA and Tukey’s test [40].

## 3. Results and Analysis

### 3.1. Single-Factor Optimization of Strawberry Wine Fermentation Conditions

**Figure 1:**
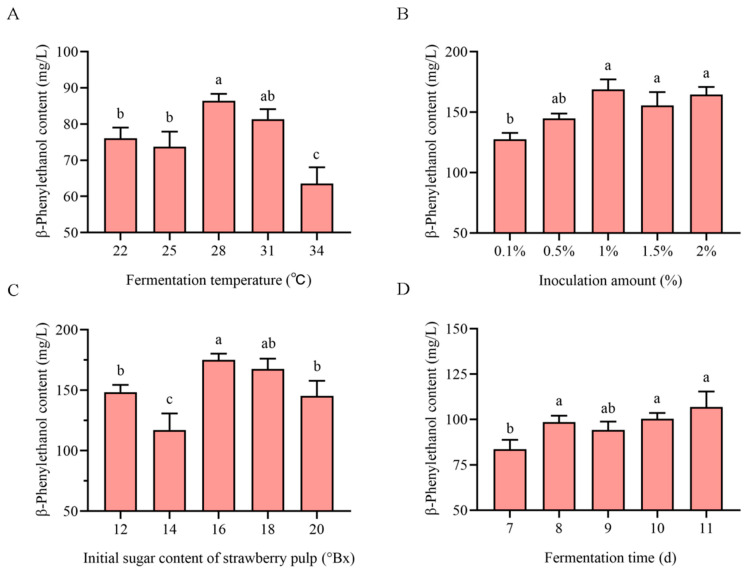
Effects of fermentation conditions on 2-phenylethanol concentration in strawberry wine fermented by *S. cerevisiae* AFRC01: (**A**) 2-phenylethanol concentration at different temperatures; (**B**) Impact of inoculation amount on 2-phenylethanol concentration; (**C**) Effect of initial °Brix on 2-phenylethanol concentration; (**D**) Influence of fermentation time on 2-phenylethanol concentration. Different superscript letters indicate significant differences (*p* < 0.05). Data are presented as mean ± standard deviation (mean ± SDs) (*n* = 3).

Optimal temperature ensures yeast viability, fermentation efficiency, and flavor metabolite synthesis. Our results demonstrate that 2-phenylethanol content in strawberry wine initially decreased and increased then decreased with rising fermentation temperatures, reaching a maximum yield of 86.420 mg/L at 28 °C (Figure 1A). The inoculation amount critically influenced flavor compound accumulation and complexity. The 2-phenylethanol content rose initially with an increase in inoculation but then declined beyond 1.0% inoculum, reaching its maximum (168.743 mg/L) at this level (Figure 1B). The initial sugar concentration significantly influenced fruit wine fermentation. Excessive sugar levels inhibited yeast metabolic activity, while insufficient sugar resulted in incomplete substrate utilization. As shown in Figure 1C, 2-phenylethanol production exhibited a biphasic response to sucrose concentration, peaking at 175.027 mg/L with 16 °Bx. The 2-phenylethanol concentrations in strawberry wine fermented for 8 days (93.63 mg/L) and 11 days (106.76 mg/L) showed no statistically significant difference (*p* > 0.05) (Figure 1D). To optimize fermentation efficiency while minimizing production time and cost, we selected 8 days as the optimal fermentation duration for subsequent experiments, so the fermentation time was not used as an indicator for further optimization. The single-factor results indicated that the optimization of fermentation conditions for strawberry wine should be selected at a temperature of 25–31 °C, an inoculum volume of 0.5–1.5%, and an initial sucrose concentration of 14–18 °Bx.

### 3.2. Response Surface Methodology (RSM) Optimization Experiments

Response surface methodology (RSM) was employed to analyze the three significant factors: fermentation temperature, inoculation amount, and initial °Brix, Details of the experimental design are provided in Table S6. Design-Expert 13 software generated the following regression equation:

Y = +171.39 + 0.4562A + 6.65B + 3.47C − 9.63AB + 7.39AC + 0.4275BC − 330.19A^2^ − 114.36B^2^ − 222.59C^2^

**Figure 2:**
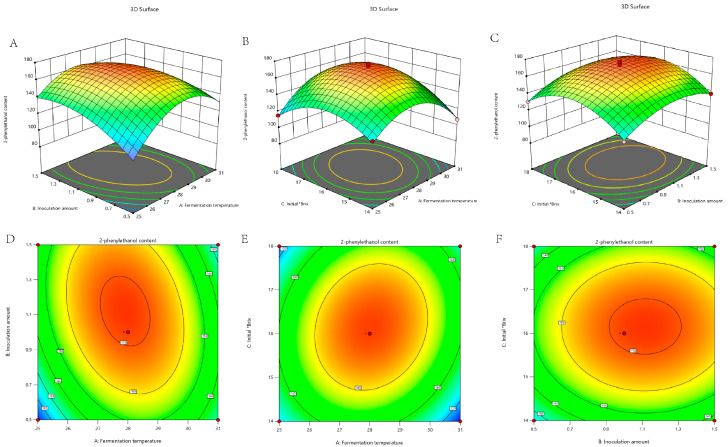
Response surface analysis results: (**A**) 3D surface plot: Fermentation temperature × inoculation amount interaction; (**B**) 3D surface plot: Fermentation temperature × Initial °Brix interaction; (**C**) 3D surface plot: Inoculum amount × Initial °Brix interaction; (**D**) Contour plot: inoculation amount and fermentation temperature interaction; (**E**) Contour plot: Initial °Brix and fermentation temperature interaction; (**F**) Contour plot: Initial °Brix and inoculum amount interaction.

The model’s statistical evaluation indicated high significance (*** *p* < 0.0001), with terms B, C, AB, AC, A^2^, B^2^, and C^2^ identified as significant model components (Table S7). This demonstrates that these factors and their interactions critically regulate 2-phenylethanol biosynthesis. In addition, the model’s R^2^ (0.9873) closely matched the adjusted R^2^ (0.9795), confirming high accuracy (Table S8). Contour plots and 3D response surfaces further demonstrated that 2-phenylethanol concentration was predominantly influenced by the interactive effects of fermentation temperature and inoculation amount (Figure 2A,B), fermentation temperature and initial °Brix (Figure 2C,D), and inoculation amount and initial °Brix (Figure 2E,F). RSM optimization yielded optimal parameters: fermentation temperature (A) = 27.959 °C, inoculation amount (B) = 0.998%, and initial °Brix (C) = 15.648 °Bx. For industrial feasibility, the operational parameters were standardized to 28 °C fermentation temperature, 1% inoculation amount, and 16 °Bx. Under these fermentation conditions, the measured 2-phenylethanol concentration was 169.924 mg/L, which closely aligns with the model-predicted value (170.049 mg/L).

### 3.3. Sensory Analysis of Strawberry Wine

**Figure 3:**
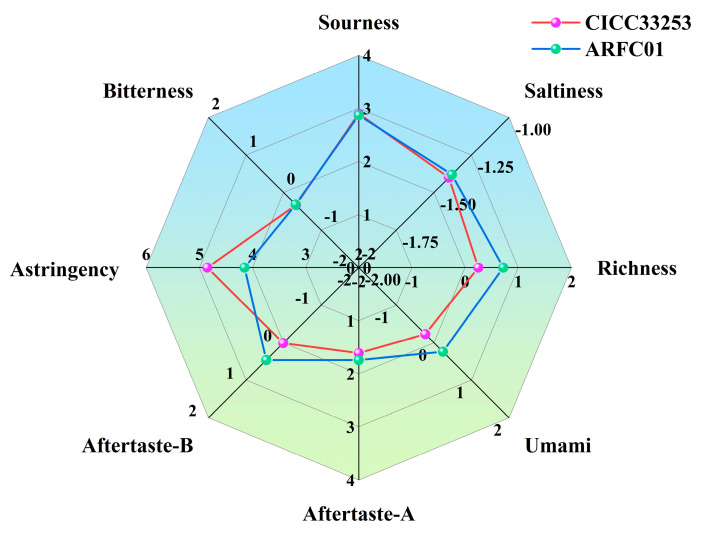
Radar chart of electronic tongue measurements: Outer labels indicate sensory attributes, with pink and green traces representing results for *S*. *cerevisiae* CICC33253 and *S*. *cerevisiae* AFRC01, respectively. Each group of tests was three replicates and data are presented as mean.

The electronic tongue analysis demonstrated distinct taste profile differences between wines fermented with *S. cerevisiae* CICC33253 and *S. cerevisiae* AFRC01 (Figure 3). Specifically, *S. cerevisiae* AFRC01 fermented wine showed significant sensory improvements, including an astringency reduction by 0.70 units; a richness enhancement of 0.45 units; and Umami increased 0.47 units. No significant changes were observed in Aftertaste-A, Sourness, Bitterness and Saltiness. These results indicate that *S. cerevisiae* AFRC01 effectively improved the sensory characteristics of strawberry wine.

### 3.4. qRT-PCR of Analysis

**Figure 4:**
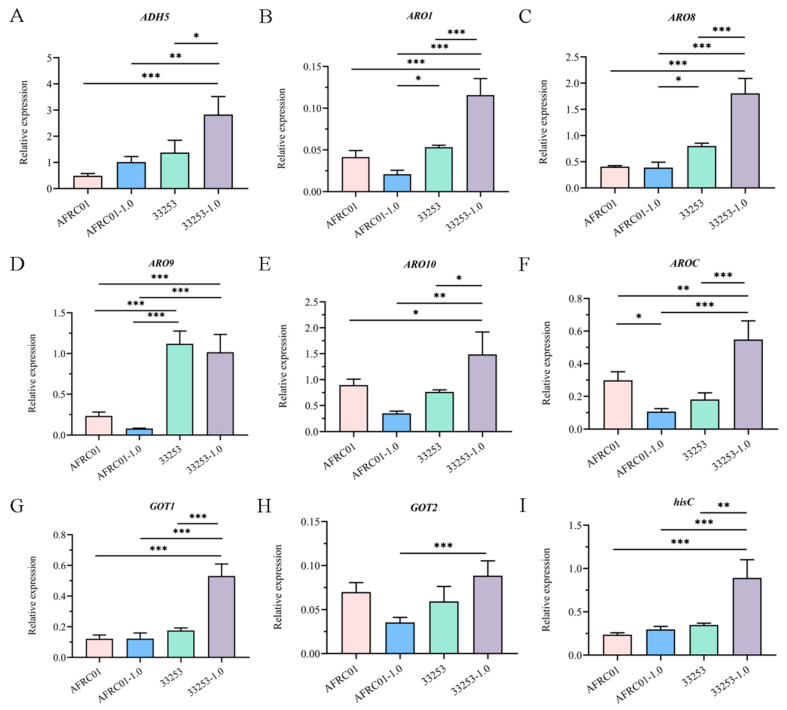
Expression pattern analysis of 2-Phenylethanol biosynthesis-related genes: (**A**–**I**) shows the expression patterns of nine genes in two strains, comparing treated (1.0 g/L 2-phenethyl alcohol) and untreated conditions. Data are presented as mean ± standard deviation (mean ± SDs) (*n* = 3). Asterisks indicate statistically significant differences among groups (* *p* < 0.05, ** *p* < 0.01, *** *p <* 0.001).

We analyzed the expression patterns of nine genes (*ADH5*, *ARO1*, *ARO8*, *ARO9*, *ARO10*, *AROC*, *GOT1*, and *GOT2*, *hisC*) related to the major pathways of 2-phenylethanol biosynthesis (Ehrlich and shikimate pathways). A comparative analysis of the same strain was performed under unstressed and stressed conditions. qRT-PCR results showed non-significant differences in the expression of the nine genes in *S*. *cerevisiae* CICC33253 compared with that in *S*. *cerevisiae* AFRC01 (Figure 4). The *ADH5*, *ARO1*, *ARO8*, *ARO10*, *AROC*, *GOT1* and *hisC* genes were significantly up-regulated in *S*. *cerevisiae* CICC33253 after 1.0 g/L 2-phenylethanol stress (*p* < 0.05). The *ARO9* and *GOT2* genes were not significantly different (*p* > 0.05). In contrast, AFRC01 showed no significant expression changes in the *ADH5*, *hisC*, *ARO1*, *ARO9*, *ARO10* and *GOT2* under identical conditions. In contrast, *S*. *cerevisiae* AFRC01 showed no significant expression changes in *ADH5*, *hisC*, *ARO1*, *ARO9*, *ARO10* and *GOT2* genes under identical conditions. These results indicate that 1.0 g/L 2-phenylethanol stress significantly up-regulated the expression of genes related to the synthesis of 2-phenylethanol by *S*. *cerevisiae* CICC33253, while the expression of genes related to the pathway for the synthesis of 2-phenylethanol by *S*. *cerevisiae* AFRC01 had no significant effect. Notably, we observed that the expression of *ARO1* and *AROC* genes in the shikimate pathway was down-regulated, and a significant difference was found in the *AROC* genes. This suggests that the cause of 2-phenylethanol resistance in *S*. *cerevisiae* AFRC01 may not be related to the correlation of genes related to the promotion of 2-phenylethanol synthesis, so we further determined the genomes of the two strains.

### 3.5. Genome Assembly and Functional Annotation Analysis

**Figure 5:**
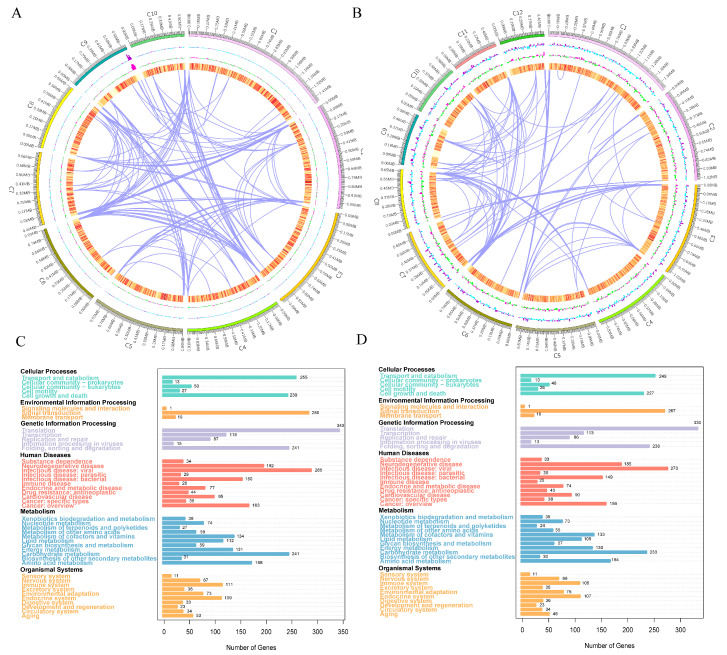
Circular genome maps and KEGG annotation results of both strains: (**A**) Circular genome map of *S*. *cerevisiae* CICC33253; (**B**) Circular genome map of *S*. *cerevisiae* AFRC01; (**C**) KEGG annotation of *S*. *cerevisiae* CICC33253; (**D**) KEGG annotation of *S*. *cerevisiae* AFRC01. In the circular genome maps, each concentric layer represents distinct functional annotation information proceeding outward to inward: genomic physical coordinates and gene distribution; GC content (blue regions indicate GC content below the genomic average); GC-skew [(G − C)/(G + C)] (green: <0, pink: >0); and Gene Duplication events. For KEGG annotations, the left axis displays functional classification with numerical values indicating annotated gene counts.

A BUSCO assessment revealed genome completeness of 97.90% and 98.00% for the two strains, with single-copy genes accounting for 91.00% and 91.40%, duplicated genes for 6.90% and 6.60%, and both fragmented and missing genes below 0.3% and 1.8%, respectively (Table S9). This indicates excellent single-copy gene coverage and demonstrates high genome assembly integrity and continuity, providing a reliable foundation for subsequent functional annotation. The basic genomic characteristics of *S. cerevisiae* CICC33253 include a genome measuring 11,872,533 bp and a GC content of 39.56% (Figure 5A), while those of *S. cerevisiae* AFRC01 include a genome length of 11,876,077 bp and a GC content of 39.61% (Figure 5B).

An analysis using the Carbohydrate-Active Enzymes database (CAZy) revealed the distribution of CAZymes between the two strains. Both *S*. *cerevisiae* AFRC01 and CICC33253 showed abundant annotations for glycoside hydrolases (*GHs*) and glycosyl transferases (*GTs*), indicating highly active carbohydrate metabolism during growth (Figure S3). A GO functional analysis demonstrated enrichment in Biological Process: CICC33253 was primarily enriched in Cellular Process (1606, 11.78%) and Metabolic Process (1591, 11.67%), while AFRC01 showed enrichment in Metabolic Process (1582, 11.73%) and Cellular Process (1581, 11.72%). For Molecular Function, both strains were predominantly enriched in binding and catalytic activity, with CICC33253 having 1762 (12.93%) and 1410 (10.34%) annotated genes, respectively, versus AFRC01’s 1792 (13.29%) and 1387 (10.28%) (Figure S4). The core functional categories, cellular components and fundamental molecular functions within the biological processes are identical between the two strains. This shared functional architecture reflects their similarity in fundamental biological characteristics. However, the proportions of genes annotated to specific categories (cellular processes, metabolic processes, rhythmic processes, and growth). These proportional variations may relate to each strain’s distinct adaptability and metabolic specificity, indicating potential divergences in their survival strategies and functional expansion. The KEGG analysis annotated 4344 genes in *S. cerevisiae* CICC33253 (Figure 5C) and 4215 in *S. cerevisiae* AFRC01 (Figure 5D). Both strains exhibited high gene counts in core metabolic pathways including carbohydrate metabolism, amino acid metabolism, and energy metabolism, indicating conserved functional capabilities for energy acquisition and material conversion. However, differential pathway enrichment was observed: signal transduction genes accounted for 6.45% in *S. cerevisiae* CICC33253 versus 6.33% in *S. cerevisiae* AFRC01; transport and catabolism for 5.87% versus 5.91%; and folding, sorting and degradation for 5.55% versus 5.65%. These differences are hypothesized to be linked to their adaptability and response mechanisms under the selective pressure of high-concentration 2-phenylethanol [29]. However, further experimental investigation is required to empirically validate these hypotheses.

### 3.6. Comparative Genomics and SNP Analysis

**Figure 6:**
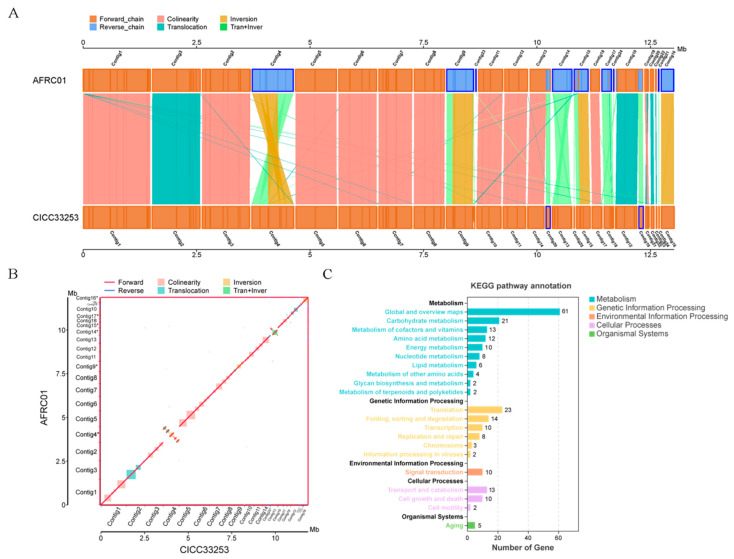
Synteny comparison and KEGG enrichment of nonsynonymous mutation-harboring genes: (**A**) Genome-wide synteny analysis between *S*. *cerevisiae* AFRC01 and *S*. *cerevisiae* CICC33253; (**B**) Two-dimensional synteny plot of *S*. *cerevisiae* AFRC01 versus *S*. *cerevisiae* CICC33253; (**C**) KEGG enrichment analysis for genes containing nonsynonymous mutations from SNP profiling. *: contig distribution.

To further investigate the mechanisms underlying *S*. *cerevisiae* AFRC01's 2-phenylethanol tolerance, we performed a synteny analysis against the *S*. *cerevisiae* CICC33253 reference genome. The synteny results (Figure 6B) demonstrate high genomic conservation between *S*. *cerevisiae* AFRC01 and *S*. *cerevisiae* CICC33253 with 178 syntenic blocks, while structural variations (SVs) including inversions, translocations, and Tran + Inver events were observed, suggesting potential associations with adaptive evolution in the strains. The synteny plot (Figure 6A) further elucidates genomic correspondence between strains, clearly visualizing contig distributions, structural features, and variation landscapes; differentially colored lines/regions denote gene orientations, functional modules, and structural variation (SV) types/loci, suggesting potential impacts on the expression regulation and functional implementation of 2-phenylethanol biosynthesis/metabolism-related genes.

Further SNP analysis identified 2824 total variants, comprising synonymous mutations (740, 26.20%), nonsynonymous mutations (483, 17.10%), stop-retain mutations (0.07%), and stop-loss mutations (0.10%) (Table S10). KEGG enrichment of the 483 nonsynonymous mutation-harboring genes (Figure 6C) annotated 239 genes, predominantly distributed across Metabolism (139), Genetic Information Processing (60), Environmental Information Processing (10), Cellular Processes (25), and Organismal Systems (5). This mutation profile reflects functional divergence between strains, with *S. cerevisiae* AFRC01 exhibiting primary metabolic pathway differentiation relative to *S. cerevisiae* CICC33253.

### 3.7. Analysis of Mutation Loci Associated with 2-Phenylethanol Metabolic Pathways

**Figure 7:**
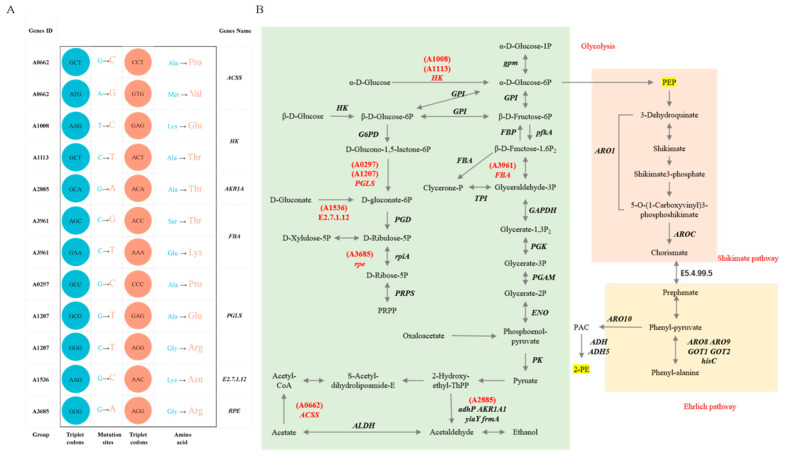
Metabolic pathway analysis of 2-Phenylethanol production in mutant yeast strain AFRC01: (**A**) mutation sites of relevant genes in the 2-Phenylethanol metabolic pathway; (**B**) diagram of 2-Phenylethanol synthesis pathway, where the red font represents the position of mutant genes in the pathway related to the synthesis of 2-Phenylethanol.

We conducted a metabolic pathway analysis focused on 2-phenylethanol biosynthesis in *S*. *cerevisiae*, primarily examining glycolysis (Ko00010) and the pentose phosphate pathway (Ko00030). We found no mutations in the genes of the major pathways (Ehrlich and shikimate pathways) for the synthesis of 2-phenylethanol, and mutations were identified in the glycolytic pathway mutations of the *ACSS*, *HK*, *AKR1A*, *FBA*, *PGLS*, *EC 2.7.1.12*, and *RPE* genes (Figure 7A and Table S11). These mutations are point mutations, and these mutation loci are uniformly positioned upstream from phosphoenolpyruvate (PEP), the core precursor for 2-phenylethanol biosynthesis. These seven mutated genes are hypothesized to be responsible for the improved tolerance of the yeast *S. cerevisiae* AFRC01 to 2-phenylethanol, as well as the ability of *S. cerevisiae* AFRC01 to continue synthesizing 2-phenylethanol despite the relatively high 2-phenylethanol conditions (Figure 7B).

## 4. Discussion

Strawberries are among the world’s most widely cultivated and highest-yielding crops [41]. However, their post-ripening shelf life is short, and they are highly perishable. This complicates storage, leading to significant waste and economic losses [42]. Processing fresh strawberries into products such as jam, juice, canned goods, and alcoholic/non-alcoholic beverages enables diversified utilization of strawberry resources [43,44,45]. Among the quality attributes influencing consumer choice, aroma is the key determinant of strawberry wine preference and value [46]. 2-phenethyl alcohol, an aroma compound in fermented foods such as wine and beer, inhibits the growth of various bacteria and fungi at high concentrations [47,48]. Therefore, using 2-phenylethanol resistant strains can enhance its production during fruit wine fermentation. The quality of fruit wine is closely related to the yeast strain used in fermentation, as yeast strains often result in more intense aromas and flavors. In the preliminary stage of this study, the *S. cerevisiae* CICC33253 strain was mutated using an MMC system to obtain *S. cerevisiae* AFRC01 that is resistant to 2-phenylethanol (3.9 g/L). In this study, the 2-phenylethanol tolerant yeast strain was used to ferment strawberry fruit wine, resulting in enhanced body and umami, and reduced astringency, indicating that this strain further enhances the flavor of strawberry fruit wine. Additionally, the quality of fruit wine is influenced by fermentation process conditions, including precise control of key parameters such as fermentation temperature, inoculum amount, and initial sugar content [12]. Temperature affects yeast growth and metabolism, thereby influencing fruit wine quality. In this study, the content of 2-phenylethanol in strawberry wine first increased and then decreased, which was related to the optimal growth temperature of *S. cerevisiae* AFRC01. At optimal temperatures, yeast growth and metabolic rates increase, leading to higher 2-phenylethanol content. However, at temperatures that are too low, yeast metabolism is inhibited, and at temperatures that are too high, yeast survival rates are affected, resulting in reduced 2-phenylethanol production. Research indicates that when the yeast inoculum is too high, the yeast’s self-replication consumes a significant amount of nutrients, while increased acid production inhibits yeast growth and reduces the flavor of the fruit wine; when the yeast inoculum is too low, incomplete fermentation results in lower ethanol production and less pronounced wine aroma [49]. In the strawberry wine studied here, the content of 2-phenylethanol also showed a trend of first increasing and then decreasing with increasing inoculation rate [50]. When the inoculation rate was 1.0%, the 2-phenylethanol content was highest. At this point, the yeast was in an environment with sufficient nutrients and adequate growth space, leading to rapid consumption of sugars during the early fermentation stage, rapid accumulation of ethanol, inhibition of yeast growth, and avoidance of incomplete fermentation caused by an insufficient inoculation rate. In this study, the production of 2-phenylethanol in strawberry wine also showed a trend of first increasing and then decreasing with the increase in initial sugar content. This indicates that when the initial sugar content is low, there is insufficient nutrient supply, leading to slow yeast growth and inadequate flavor development in the fermented wine. Conversely, when the initial sugar content is too high, it inhibits yeast proliferation and increases the occurrence of side reactions, resulting in a decline in the aroma and taste of the wine [51]. Therefore, different conditions have a significant impact on the yield of 2-phenylethanol. In terms of selecting optimization indicators for fruit wine fermentation processes, most studies focus on alcohol content, sensory evaluation, or a combination of both, while research using 2-phenylethanol as an evaluation indicator for fermented fruit wine is relatively scarce. Our study provides a reference for enhancing the flavor of fermented strawberry fruit wine.

Currently, no universally accepted method exists for defining *S. cerevisiae* tolerance to 2-phenylethanol stress. Typically, yeast tolerance is assessed through three parameters: growth inhibition, cellular activity, and 2-phenylethanol production capacity [52]. The maximum 2-phenylethanol concentration permitting stable yeast growth defines its tolerance threshold. We monitored the optical density (OD) changes in two strains under varying 2-phenylethanol concentrations in liquid medium to evaluate growth. At 1.0 g/L, strain CICC33252 maintained stable growth, whereas growth ceased at 1.5 g/L. Consequently, this study applied 1.0 g/L 2-phenylethanol stress to both strains to quantify the expression of genes involved in its biosynthesis. A qRT-PCR analysis revealed significant upregulation (*p* < 0.05) of *ADH5*, *ARO10*, *ARO1*, *ARO8*, *AROC*, and *GOT1* in *S. cerevisiae* CICC33253. These results demonstrate that under high 2-phenylethanol stress, *S. cerevisiae* CICC33253 accelerates phenylalanine synthesis/metabolism rather than producing 2-phenylethanol de novo. In contrast, *S. cerevisiae* AFRC01 exhibited no significant expression differences for eight of the nine genes under identical stress conditions, confirming its 2-phenylethanol tolerance through transcriptional non-responsiveness. Genomic analyses similarly revealed the molecular basis of *S. cerevisiae* AFRC01’s tolerance advantage. Comparative genomics showed that specific mutations in the key glycolysis genes HK and FBA in *S. cerevisiae* AFRC01 sustainably enhance PEP supply, thereby supporting steady-state operation of the manganic acid pathway. This structural adaptation fully explains the phenotypic differences: while *S. cerevisiae* CICC33253 stagnated at 2-phenylethanol concentrations above 1.0 g/L, *S. cerevisiae* AFRC01 maintained growth under 2-phenylethanol stress. The mutation pre-adapted *S. cerevisiae* AFRC01’s basal metabolism to the stress, eliminating the energy cost of initiating stress gene expression and resulting in transcriptional silencing at 1.0 g/L. Furthermore, functional annotation differences reflect *S. cerevisiae* AFRC01’s tolerance: shifts in gene proportions for signal transduction and degradation pathways suggest reduced requirements for environmental sensing and protein turnover, aligning with the metabolic stability needed for a tolerant phenotype. Crucially, the mutation-directed increase in PEP flux provided sufficient precursors for 2-phenylethanol synthesis, directly correlating with its superior fermentation performance. These findings offer a novel strategy for improving 2-phenylethanol tolerance and synthesis. Unlike the traditional overexpression or knockout of single synthesis genes, targeted optimization of precursor supply enhances both stress tolerance and synthesis efficiency, providing new insights for the rational design of industrial strains.

The synthesis of 2-phenylethanol primarily involves the embden meyerhof paras pathway, the pentose phosphate pathway, the shikimate pathway, and the Ehrlich pathway. Within these synthetic pathways, there are numerous key enzyme genes and complex feedback inhibitions. Genetic modification of strains at the molecular biology level can significantly increase 2-phenylethanol production [53]. Currently, most research focuses on overexpressing or knocking out genes related to 2-phenylethanol synthesis to alter the synthesis or metabolic pathways of 2-phenylethanol [54,55]. Innovative studies have also begun to enhance precursor supply through metabolic engineering. Two important precursors in the shikimate pathway are PEP and 4-phosphate erythrulose (E4P). By increasing the precursor E4P through metabolic engineering, 2-phenylethanol production can be enhanced [56]. Comparative genomic results indicate that the mutated genes in the *S. cerevisiae* AFRC01 were concentrated in the glycolytic pathway; specifically, the *HK* gene encoding hexokinase and the *FBA* gene encoding fructose-1,6-bisphosphate aldolase were mutated. Hexokinase serves as the first committed enzyme in the glycolytic pathway, catalyzing the phosphorylation of hexoses such as glucose, and plays pivotal roles in energy metabolism, cellular growth, and apoptotic regulation [57]. Mutations in the *HK* gene in *S. cerevisiae* AFRC01 may redirect the glycolytic pathway to produce large amounts of the intermediate product PEP required by the manganic acid pathway. Similarly, mutations in FBA, a key glycolytic enzyme, could alter metabolic partitioning to enhance flux toward PEP synthesis. Collectively, these adaptations may accelerate 2-phenylethanol production under high-stress conditions. However, this proposed mechanism is still hypothetical and lacks experimental validation for further analysis, such as metabolic flux analyses.

## 5. Conclusions

The optimized fermentation parameters for 2-phenylethanol production in strawberry wine using *S. cerevisiae* AFRC01 were determined as follows: 28 °C fermentation temperature, 1.0% inoculum size, 16 °Bx initial sugar concentration, and 8 day fermentation period. Under these conditions, the 2-phenylethanol concentration of strawberry wine reached 170.06 mg/L and was accompanied by an enhanced flavor profile. The 2-phenylethanol tolerance of *S. cerevisiae* AFRC01 is associated with glycolytic pathway modifications affecting PEP content, a key precursor in 2-phenylethanol biosynthesis. While this study demonstrates the industrial potential of *S. cerevisiae* AFRC01 for flavor-enhanced strawberry wine production, further research is required to validate strain genetic stability and consistency under industrial scale fermentation conditions.

## Data Availability

The original contributions presented in the study are included in the article, further inquiries can be directed to the corresponding author.

## Supplementary Materials

The following supporting information can be downloaded at: https://www.mdpi.com/article/10.3390/foods14173043/s1, Table S1: Screening test program for influencing factors of strawberry wine fermentation process; Table S2: Factors and levels of response surface test design; Table S3: Taste test conditions; Table S4: Primer sequence; Table S5: Ct values of RT-qPCR; Table S6: Experimental design and result analysis of response surface; Table S7:ANOVA results of response surfaces; Table S8: Model fitness analysis; Table S9: BUSCO statistics; Table S10: Results of SNP analysis; Table S11: Relevant mutation sites in the 2-phenylethanol metabolic pathway; Figure S1: Standard curve of 2-phenylethanol; Figure S2: Total RNA extraction and primer specificity analysis; Figure S3: Statistical chart of CAZy functional classifications and corresponding gene numbers; Figure S4: GO functional classification: (A) *S. cerevisiae* CICC33253; (B) *S. cerevisiae* AFRC01. The red, green and blue sections respectively represent Biological Processes, Cellular Components and Molecular Functions.
