# Supplementary material for: Fermentation Process Optimization of Strawberry Wine Using the 2-Phenylethanol Tolerant Saccharomyces cerevisiae AFRC01 and Comparative Genomic Analysis
Source: Foods. 2025 Aug 29;14(17):3043. doi: 10.3390/foods14173043 (PMC12427807; doi:10.3390/foods14173043)
Supplement: Supplementary file 1 [file foods-14-03043-s001.zip › foods-3825917-supplementary.pdf]

Supplementary Table S1 Screening test program for influencing factors of strawberry wine fermentation process

Sample	Fermentation temperature (℃)	Inoculation size (%)	Initial °Brix (°Bx)	Fermentation time (d)
Fermentation temperature	22, 25, 28, 31, 34	28	28	28
Inoculation size	1.0	0.1, 0.5, 1.0, 1.5, 2.0	1.0	1.0
Initial Brix	16	16	12, 14, 16, 18, 20	16
Fermentation time	8	8	8	7, 8, 9 10, 11

Supplementary Table S2 Factors and levels of response surface test design

Sample	Fermentation temperature （℃）	Inoculation size (%)	Initial °Brix （°Bx）
-1	25	0.5	14
0	28	1.0	16
1	31	1.5	18

Supplementary Table S3 Taste test conditions

Sequence	Time
Cleaning solution 1（sec）	90
Cleaning solution 2（sec）	120
Cleaning solution 3（sec）	120
Cleaning solution	30 sec、20 times：0.5、0.5、0.5、0.5、0.5、0.5、0.5、0.5 mv
Cleaning solution（sec）	30
Cleaning solution 4（sec）	3
Cleaning solution 5（sec）	3
Cleaning solution（sec）	30

Supplementary Table S4 Primer Sequence

Gene name	Primer sequence （5’-3’）	Amplicon size （bp）	Prime efficiency （%）
UBC6 （Gene ID: 856837）	F: GGACCTGCGGATACTCCTTAC<br>R: TAATCGTGTGTTGGGCTTGA	132	103.98
ADH5	F: TATCACTCACAGGAGACCAT<br>R: ATCAGACTTCAAGACGGTTC	91	113.81
ARO8	F: CAGGGACAAGATTGGGTTGG<br>R: ACCTGGATAGCGTAGCGTTG	129	95.68
ARO9	F: CTGACTACTGAACGGTATT<br>R: TAGCAACGATGAAGGATAA	136	102.21
ARO10	F: ACCACTCAAACGCTCACATC<br>R: CACCGTCACCTTCAAACAAA	81	96.45
ARO1	F: TTCTAAGTCCATCTCCAAT<br>R: TACCGTCTCACCATTATC	163	110.17
AROC	F: ATGCTATGTTGTCCATTC<br>R: TCACCATTAGAGATACCA	175	106.28
GOT1	F: GCAGGAAGACAGAGTAAT<br>R:CCAAGTAGGCTTAGACAA	121	103.54
GOT2	F: AGCAAGAGAACAATAAGAAC<br>R: TAAACCCTGATAAGCCATAT	158	109.67
hisC	F: TACTTCACCAGGTAATCCAA<br>R: GCTTCATCAACAACGACTAA	99	102.21

Supplementary Table S5 Ct values of RT-qPCR

	UBC6	*ADH5*	*ARO8*	*ARO9*	*ARO10*	*ARO1*	*AROC*	*GOT1*	*GOT2*	*hisC*
	26.60	28.92	29.19	30.33	28.36	32.30	30.02	31.01	32.07	30.15
AFRC01	26.14	28.94	29.44	30.30	28.14	32.72	29.65	30.88	31.76	30.26
	26.25	29.38	29.39	29.85	28.13	32.93	29.73	31.41	31.84	29.98
	26.58	26.18	27.57	30.18	27.88	31.79	29.48	29.20	31.11	28.07
AFRC01-1.0	26.17	26.07	27.92	29.73	27.50	32.01	29.51	29.65	31.12	27.92
	26.26	26.43	27.41	29.68	27.89	31.70	29.44	29.06	30.94	27.96
	26.63	25.13	25.62	25.01	25.68	29.54	27.52	27.88	29.74	26.89
33253	26.12	24.28	25.51	25.09	25.65	29.45	27.97	27.60	28.88	26.66
	26.24	25.23	25.71	25.31	25.71	29.57	27.84	27.93	29.59	26.90
	26.47	24.26	25.66	26.42	25.74	29.66	27.45	27.48	29.95	26.55
33253-1.0	26.36	24.15	25.57	26.40	26.04	29.44	27.10	27.26	29.98	26.72
	26.16	24.31	25.76	26.69	26.13	29.79	27.63	27.54	30.12	26.81

Supplementary Table S6 Experimental design and result analysis of response surface

		Factor1	Factor2	Factor3	Response1
Std	Run	A: Fermentation Temperature (℃)	B: Inoculation Amount (%)	C: Initial Brix (°Bx)	2-phenylethanol content (mg/L)
1	15	25	0.5	16	109.18
2	5	31	0.5	16	133.16
3	14	25	1.5	16	139.79
4	8	31	1.5	16	125.23
5	13	25	1.0	14	124.80
6	3	31	1.0	14	107.14
7	10	25	1.0	18	115.30
8	2	31	1.0	18	127.19
9	16	28	0..5	14	122.92
10	6	28	1.5	14	137.34
11	17	28	0.5	18	130.69
12	7	28	1.5	18	146.82
13	4	28	1.0	16	165.23
14	1	28	1.0	16	175.86
15	9	28	1.0	16	169.63
16	12	28	1.0	16	174.05
17	11	28	1.0	16	172.16

Supplementary Table S7 ANOVA results of response surfaces

Source	Sum of squares	Mean square	F-value	P-value	Significant
Model	8614.34	957.15	60.42	<0.0001	***
A- Fermentation Temperature	1.67	1.67	0.1051	0.7552	--
B- Inoculation Amount	354.18	354.18	22.36	0.0021	**
C- Initial Brix	96.60	96.60	6.10	0.0429	*
AB	371.33	371.33	23.44	0.0019	**
AC	218.30	218.30	13.78	0.0075	**
BC	0.7310	0.7310	0.0461	0.8360	--
A^2	3837.75	3837.75	242.27	<0.0001	***
B^2	867.71	867.71	54.78	0.0001	***
C^2	2148.29	2148.29	135.61	<0.0001	***
Residual	110.89	15.84	--	--	--
Lack off it	42.20	14.07	0.8190	0.5472	--
Pure Error	68.69	17.17	--	--	--
Cor Total	8725.22	--	--	--	--

Note: * denotes a significant difference (P < 0.05), ** denotes a highly significant difference (P < 0.01), and * indicates**

an extremely significant difference (P < 0.001).

Supplementary Table S8 Model fitness analysis

Std.dev	mean	C.V.%	R^2	AdjustedR^2	PredictedR^2	AdeqPrecision	Std.Dev.
5.010	139.79	2.85	0.9873	0.9710	0.9103	20.6987	3.98

Supplementary Table S9 BUSCO statistics

Sample	C	S	D	F	M	N
CICC33253	98.0%	91.4%	6.6%	0.3%	1.7%	290
AFRC01	97.9%	91.0%	6.9%	0.3%	1.8%	290

Note: C: Complete; S: single-copy; D: duplicated; F: Fragmented; M: Missing ; N: Total number of gene entries tested.

Supplementaryt Table S10 Results of SNP analysis

Type	SNP Number
Start_syn	0
Stop_syn	2
Start_nonsyn	0
Stop_nonsyn	3
Premature_stop	0
Synonymous	740
Nonsynonymous	483
Total_CDS_SNP	1,228
Intergenic	1,596
Total_SNP	2,824

Supplementary Table S11 Relevant mutation sites in the 2-phenylethanol metabolic pathway

Genes ID	Name	Gene location	Mutation sites	Triplet codons	amino acid
A0662	*ACSS*	Contig13_65843	G<->C	GCT<->CCT	Ala <-> Pro
A0662	*ACSS*	Contig13_66062	A<->G	ATG<->GTG	Met <-> Val
A1008	*HK*	Contig16_117628	T<->C	AAG<->GAG	Lys <-> Glu
A1113	*HK*	Contig17_123183	C<->T	GCT<->ACT	Ala <-> Thr
A2885	*AKR1A*	Contig4_712382	G<->A	GCA<->ACA	Ala <-> Thr
A3961	*FBA*	Contig7_239128	C<->G	AGC<->ACC	Ser <-> Thr
A3961	*FBA*	Contig7_239381	C<->T	GAA<->AAA	Glu <-> Lys
A0297	*PGLS*	Contig11_471573	G<->C	GCC<->CCC	Ala <-> Pro
A1207	*PGLS*	Contig18_149352	G<->T	GCG<->GAG	Ala <-> Glu
A1207	*PGLS*	Contig18_149491	C<->T	GGG<->AGG	Gly <-> Arg
A1536	*E2.7.1.12*	Contig1_917553	G<->C	AAG<->AAC	Lys <-> Asn
A3685	*RPE*	Contig6_248947	G<->A	GGG<->AGG	Gly <-> Arg

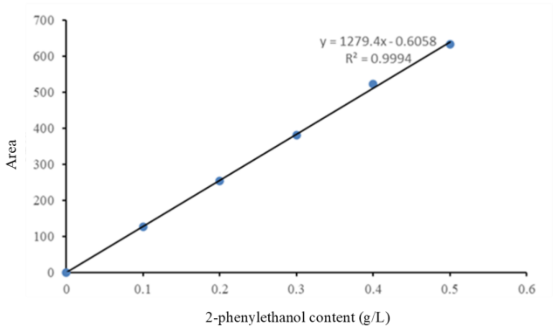

Supplementary Figure S1. Standard curve of 2-phenylethanol: The horizontal axis represents the 2-phenylethanol content, and the vertical axis represents the peak area.

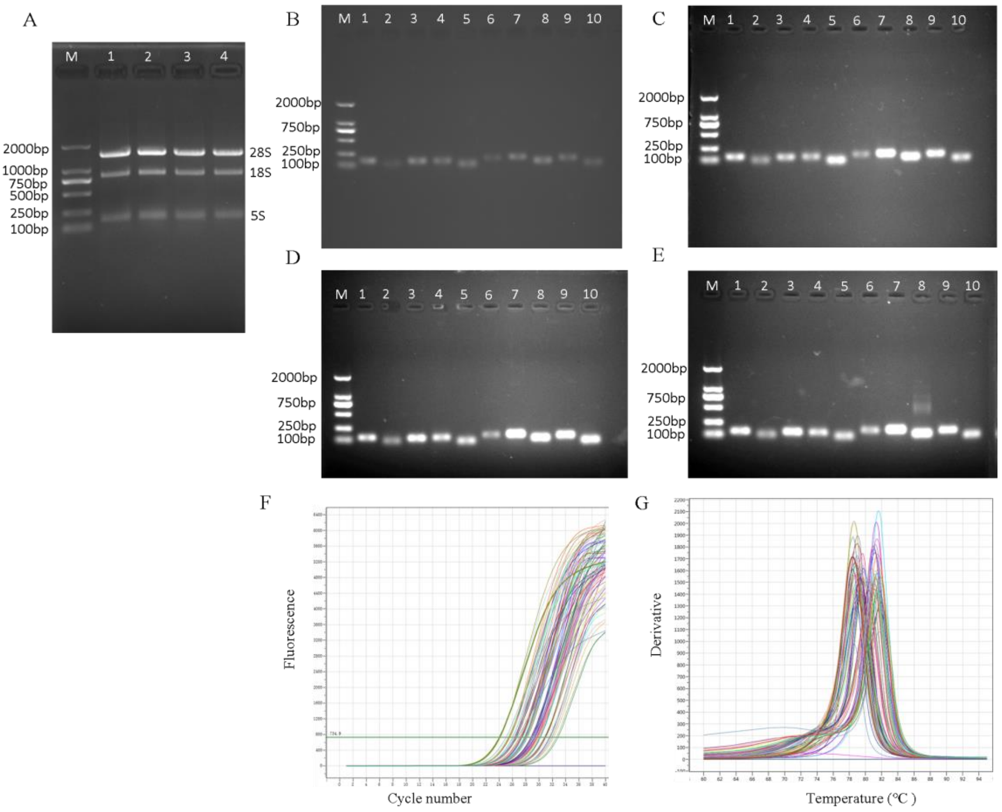

Supplementary Figure S2. Total RNA extraction and primer specificity analysis: (A) RNA electrophoresis results: the 33253, 33253-1.0, AFRC01 and AFRC01-1.0 samples correspond to lanes 1-4; (B)- (E) The 33253, 33253-1.0, AFRC01, AFRC01-1.0 samples PCR result; Lanes 1-10 correspond to PCR amplicons of *UBC6*, *ADH5*, *ARO8*, *ARO9*, *ARO10*, *ARO1*, *AROC*, *GOT1*, *GOT2*, and *hisC* genes; (F) Amplification Curve; (G) Dissociation Curve.

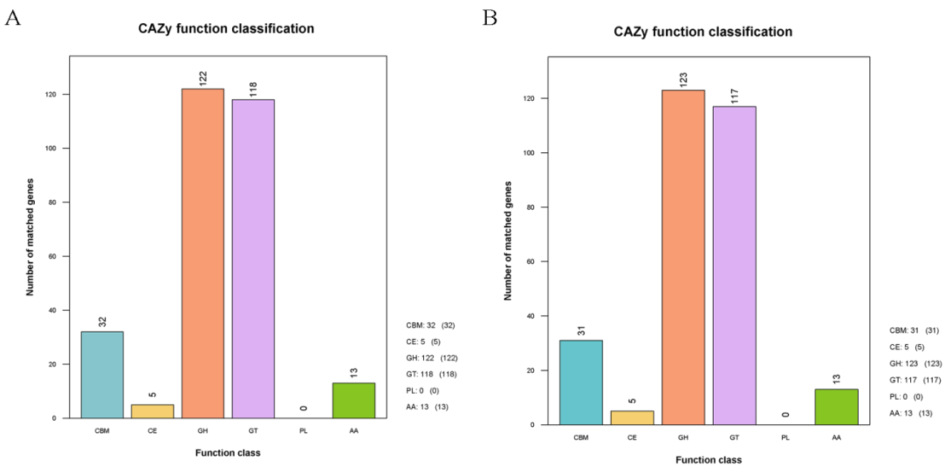

Supplementary Figure S3. Satistical chart of CAZy functional classifications and corresponding gene numbers: (A) *S. cerevisiae* CICC33253; (B) *S. cerevisiae* AFRC01. GH: Glycoside Hydrolases, GT: Glycosyl Transferases, CBM: Carbohydrate-binding modules, AA: Auxiliary Activities, CE: Carbohydrate Esterases.

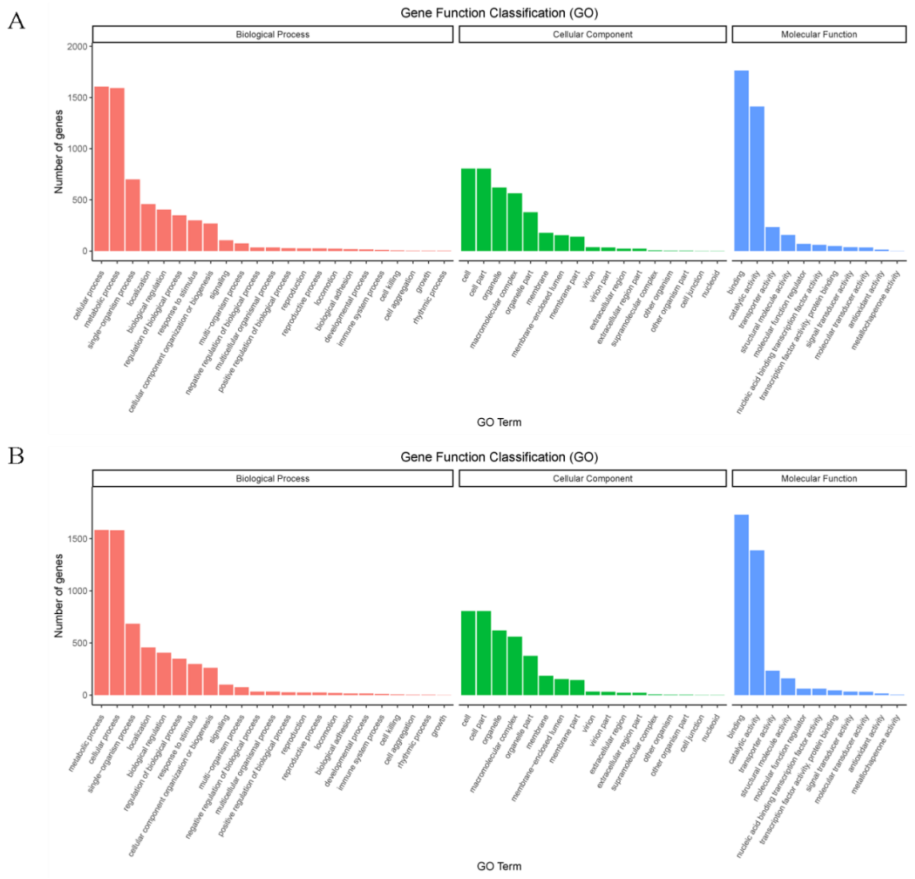

Supplementary Figure S4. GO functional classification**:** (A) *S. cerevisiae* CICC33253; (B) *S. cerevisiae* AFRC01. The red, green and blue sections respectively represent Biological Processes, Cellular Components and Molecular Functions.
